# IL-37 Inhibits Inflammasome Activation and Disease Severity in Murine Aspergillosis

**DOI:** 10.1371/journal.ppat.1004462

**Published:** 2014-11-06

**Authors:** Silvia Moretti, Silvia Bozza, Vasilis Oikonomou, Giorgia Renga, Andrea Casagrande, Rossana G. Iannitti, Matteo Puccetti, Cecilia Garlanda, Soohyun Kim, Suzhao Li, Frank L. van de Veerdonk, Charles A. Dinarello, Luigina Romani

**Affiliations:** 1 Department of Experimental Medicine and Biochemical Sciences, University of Perugia, Perugia, Italy; 2 Istituto Superiore di Sanità, Roma, Italy; 3 Humanitas Clinical and Research Center, Rozzano, Milan, Italy; 4 Department of Medicine, University of Colorado Denver, Aurora, Colorado, United States of America; 5 Department of Medicine, Radboud University Medical Centre, Nijmegen, The Netherlands; University of Wisconsin-Madison, United States of America

## Abstract

Since IL-37 transgenic mice possesses broad anti-inflammatory properties, we assessed whether recombinant IL-37 affects inflammation in a murine model of invasive pulmonary aspergillosis. Recombinant human IL-37 was injected intraperitoneally into mice prior to infection and the effects on lung inflammation and inflammasome activation were evaluated. IL-37 markedly reduced NLRP3-dependent neutrophil recruitment and steady state mRNA levels of IL-1β production and mitigated lung inflammation and damage in a relevant clinical model, namely aspergillosis in mice with cystic fibrosis. The anti-inflammatory activity of IL-37 requires the IL-1 family decoy receptor TIR-8/SIGIRR. Thus, by preventing activation of the NLRP3 inflammasome and reducing IL-1β secretion, IL-37 functions as a broad spectrum inhibitor of the innate response to infection-mediated inflammation, and could be considered to be therapeutic in reducing the pulmonary damage due to non-resolving *Aspergillus* infection and disease.

## Author Summary

IL-37, firstly identified by *in silico* research in the year 2000, is a member of the IL-1 family. The biological properties of IL-37 are mainly those of down-regulating inflammation in models of septic shock, chemical colitis, cardiac ischemia and contact dermatitis. Whether and how IL-37 down-regulates the inflammation of infection, and its consequences, is not known. We observed that IL-37 limits inflammation and disease severity in murine invasive aspergillosis, an infection model in which cytokines of the IL-1 family have important roles. However, given that IL-1R1-deficient or caspase 1-deficient mice are resistant to lung inflammation during infection and that IL-1 signaling could drive the differentiation of antifungal inflammatory Th17 cells, the pro-inflammatory properties of IL 1-induced inflammation in aspergillosis is potentially dangerous for the host. IL-37 markedly reduced NLRP3-dependent neutrophil recruitment and steady state mRNA levels of IL-1β production and mitigated lung inflammation and damage in a relevant clinical model, namely aspergillosis in mice with cystic fibrosis. The anti-inflammatory activity of IL-37 requires the IL-1 receptor family decoy TIR-8/SIGIRR. Thus, IL-37 functions as a broad spectrum inhibitor of infection-mediated inflammation, and could be considered to be therapeutic in reducing the pulmonary damage due to non-resolving *Aspergillus* infection and disease.

## Introduction

IL-37 is a member of the IL-1 family of ligands discovered by computational cloning and previously termed IL-1 family member 7 [1]. Five different splice variants of IL-37 have been described [2], [3]. The major splice variant is IL-37b [4] and, similar to most members of the IL-1 family, lacks a clear signal peptide. The precursor form is a ∼30-kDa molecular mass protein that shares critical amino acid residues with IL-18 [5]. In fact, IL-37 binds to the IL-18 receptor [6] as well as the IL-18 binding protein [7]. The first indication that IL-37 possessed anti-inflammatory properties was observed with the combination of IL-37 plus IL-18 binding protein [7]. Staining for IL-37 of human PBMC shows a granular pattern in close proximity to the Golgi and endoplasmic reticulum, a pattern, which suggests translocation via secretory vesicles [6], [7]. IL-37 translocates to the nucleus and reduces LPS-induced cytokines. The nuclear translocation of IL-37 requires caspase-1 activity as assessed by caspase-1 inhibitors [8] or by mutation of the caspase-1 recognition aspartic acid in the IL-37 precursor [9]. IL-37 exerts anti-inflammatory effects by suppressing innate immune responses through attenuating the production of inflammatory cytokines induced by TLR agonists, IL-1 and tumor necrosis factor (TNF) [8], [10].

IL-37 specific mRNA has been detected in a variety of normal tissues and tumors in humans, where it is up-regulated by inflammatory stimuli and cytokines [10]–[12], a finding suggesting that IL-37 mediates a negative feedback mechanism to curb excessive inflammation. Although a mouse homologue has not yet been identified, IL-37b has been reported to act as an inhibitor of inflammation in mice, a function achieved by inhibition of dendritic cell activation on the cellular level and by interaction with Smad3 and modulation of kinase checkpoints on the molecular level [10]. Transgenic mice expressing human IL-37 on haematopoietic cells were protected from chemically-induced colitis [13] and from local and systemic inflammation in ConA-induced hepatitis and LPS challenge [14]. These results place IL-37 within the portfolio of classical anti-inflammatory cytokines, such as IL-10 and TGF-β [15].

Cytokines of the IL-1 family have important roles for antifungal host defense in the lung [16] and IL-1 gene cluster polymorphisms has been associated with susceptibility to aspergillosis in hematologically suppressed patients [17]. IL-1α, IL-1β and IL-18 are induced in alveolar macrophages, blood monocytes and neutrophils in response to *Aspergillus* in mice and humans. In turn, these cytokines activate the release of other pro-inflammatory cytokines such as TNF-α and IL-6, and induce antifungal Th17 responses [18]–[20]. In contrast to conidia, *Aspergillus* hyphae also induced NLRP3 inflammasome assembly, caspase-1 activation and IL-1β release from a human monocyte cell line [21]. However, given that IL-1R1-deficient or caspase 1-deficient mice are resistant to lung inflammation during aspergillosis [20], [22] and that IL-1 signaling could drive the differentiation of antifungal inflammatory Th17 cells [20], [23], the proinflammatory properties of IL-1-induced inflammation in aspergillosis is potentially dangerous for the host. Therefore, in order to reduce inflammation in this model, we assessed whether IL-37 would dampen inflammation in experimental pulmonary inflammatory aspergillosis.

## Results

### IL-37 reduces inflammatory cell recruitment in mice with aspergillosis

**Figure 1:**
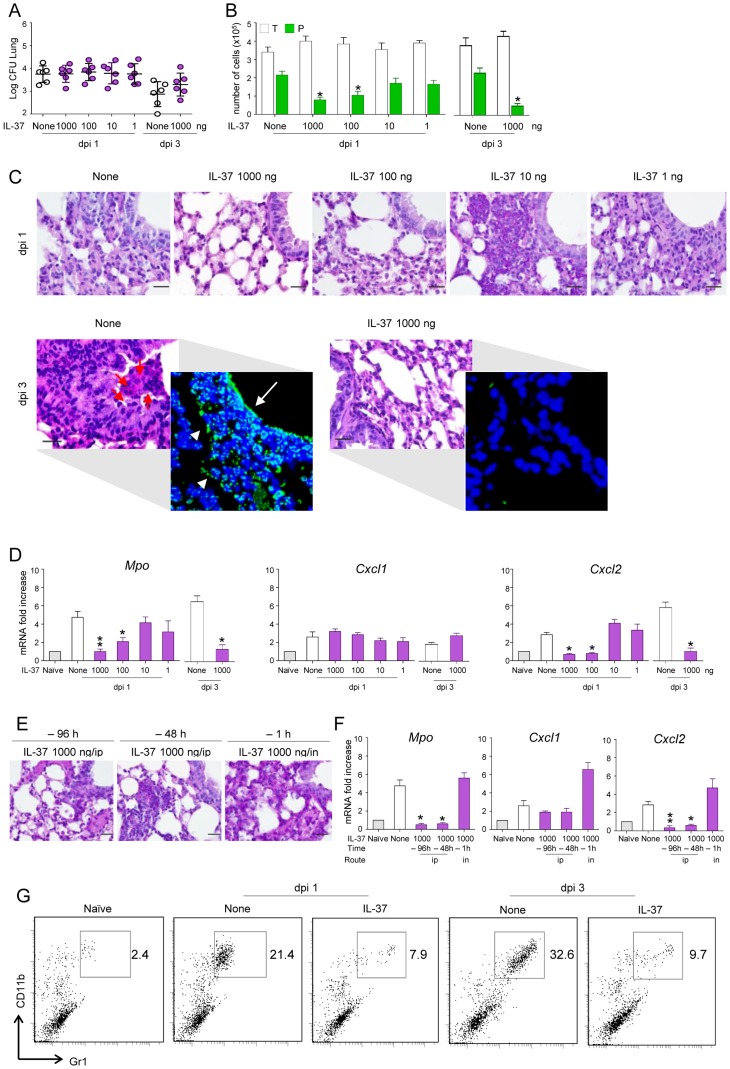
IL-37 reduces inflammatory cell recruitment in mice with inflammatory aspergillosis. C57BL/6 mice were infected intranasally (in) with *A. fumigatus* and pretreated one time with different doses IL-37 administered intraperitoneally (ip) at different times before the infection. Mice were assessed for: (**A**) fungal growth (Log_10_ CFU, mean±SD) in the lungs at 1 and 3 days post-infection (dpi); (**B**) BAL fluid morphometry [number of total (T) cells and polymorphonuclear neutrophils (P) upon May Grunwald Giemsa staining. Values represent the mean±SD of three mice per group and are representative of 3 independent experiments]; (**C**) lung histology (periodic acid-Schiff and, in the inset, TUNEL staining). Red arrows indicate PMN and white arrows indicate increased deposition of DNA on lung parenchyma (in TUNEL-stained sections). Scale bars, 25 µm; (**D**) myeloperoxidase (*Mpo*), *Cxcl1* and *Cxcl2* mRNA expression by RT-PCR on total lung cells; (**E**) lung histology (periodic acid-Schiff staining, scale bars, 25 µm) and (**F**) *Mpo*, *Cxcl1* and *Cxcl2* mRNA expression (RT-PCR on total lung cells) in mice pretreated with IL-37 given ip or in, at different hours before the infection. (**G**) Numbers of CD11b/Gr1–positive cells were assessed by flow cytometry of total lung cells from LPS-treated mice. Data are representative (histology) or pooled from three experiments. *P<0.05,**P<0.01, treated *vs* untreated (None) mice. Naïve, uninfected and untreated mice.

We first assessed the impact of the recombinant IL-37 precursor (hereafter referred to as IL-37) on lung inflammation. The IL-37 precursor was administered intraperitoneally once either 96, 48 or 1 hour before intranasal infection with live *A. fumigatus* conidia. Mice were monitored 1 and 3 days after the infection for BAL morphometry, inflammatory cell recruitment and expression of myeloperoxidase (*Mpo*) and chemokines. Although not affecting the fungal burden in the lung (Figure 1A), IL-37 administered 1 h before the infection at the dosage of 1000 and 100 ng/mouse (50 and 5 µg/kg, respectively) reduced BAL neutrophilia (Figure 1B), neutrophil influx in the lung (Figure 1C), lung damage (Figure 1C, insets) and lung expression of *Mpo* and *CxCl2*, an essential mediator of host defense against *A. fumigatus* in mice [24] and humans [25]. *CxCl1* expression was instead unaffected (Figure 1D). These reductions were observed as early as 1 day after the infection and appears to be long-lasting, being still present at 3 days post-infection, a time at which BAL neutrophilia (Figure 1B), lung neutrophilic infiltration (Figure 1C) and *Mpo* and *CxCl2* expression (Figure 1D) were drastically reduced. Accordingly, IL-37 was effective when administered 96 or 48 hours before the infection (Figure 1E and F). The effects was strictly dependent on the route of administration, being lost upon local intranasal injection (Figure 1E and F). Of interest, IL-37 was also effective in dampening inflammation when administered after the infection (Figure S1). Similar to *Aspergillus*, IL-37 also reduces neutrophil infiltration in mice treated with LPS (Figure 1G), a finding indicating that IL-37 inhibits TLR-dependent neutrophil recruitment in lung infections as reported previously [10].

### IL-37 impairs inflammasome activation in mice with aspergillosis

**Figure 2:**
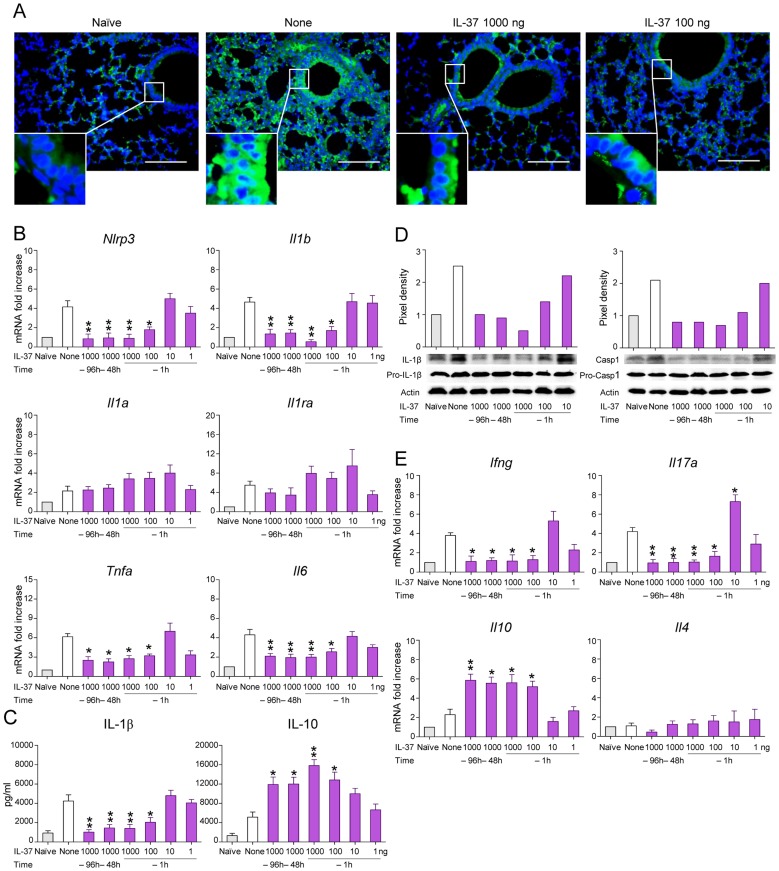
IL-37 impairs inflammasome activation in mice with aspergillosis. C57BL/6 mice were infected intranasally with *A. fumigatus* and treated intraperitoneally with recombinant IL-37 precursor, at the indicated doses, 96, 48 and 1 hour before the infection. (**A**) NLRP3 expression in the lung by immunofluorescence staining with anti-CIAS1/Nlrp3 antibody. In the insets, positive staining of epithelial cells. Nuclei were counterstained with DAPI. Scale bars, 100 µm. (**B, E**) Gene expression on total lung cells by RT-PCR. (**C**) Cytokine production (ELISA) on lung homogenates. (**D**) Immunoblot analysis on whole lung lysates of IL-1β and Caspase 1 using rabbit specific antibodies and rabbit anti-actin. Goat anti-rabbit IgG-HRP were used as secondary antibody. Corresponding pixel density ratio was normalized against actin. Assays were done a day after the infection. Data are representative (immunoblotting) or pooled from three experiments. *P<0.05, **P<0.01, treated *vs* untreated (None) mice. Naïve, uninfected and untreated mice.

As uncontrolled IL-1β promotes detrimental neutrophil-dependent inflammation during aspergillosis [20], we examined whether IL-37 pretreatment affects the level of IL-1β production and inflammasome activation. As shown in Figure 2 by immunohistochemistry (Figure 2A) and RT-PCR (Figure 2B), lung NLRP3 expression increased after the infection in both epithelial and in the recruited inflammatory cell compartment. IL-37, at 1000 and 100 ng/mouse, greatly reduced *Nlrp3* steady state mRNA levels in the lungs. IL-1β was also decreased by IL-37, as revealed by RT-PCR (Figure 2B), ELISA (Figure 2C) and pro-IL-1β and caspase-1 cleavage by immunoblotting (Figure 2D). IL-37 did not inhibit the expression of *Il1a* (Figure 2B), did not change that of *Il1ra* (Figure 2B), known to inhibit inflammasome activation [26], and only partially reduced the expression *Tnfa* and *Il6* (Figure 2B). IL-37 also reduced the expression of *Il17a* and *Ifng* (Figure 2E) and greatly increased that of *Il10* (Figure 2C and E).

**Figure 3:**
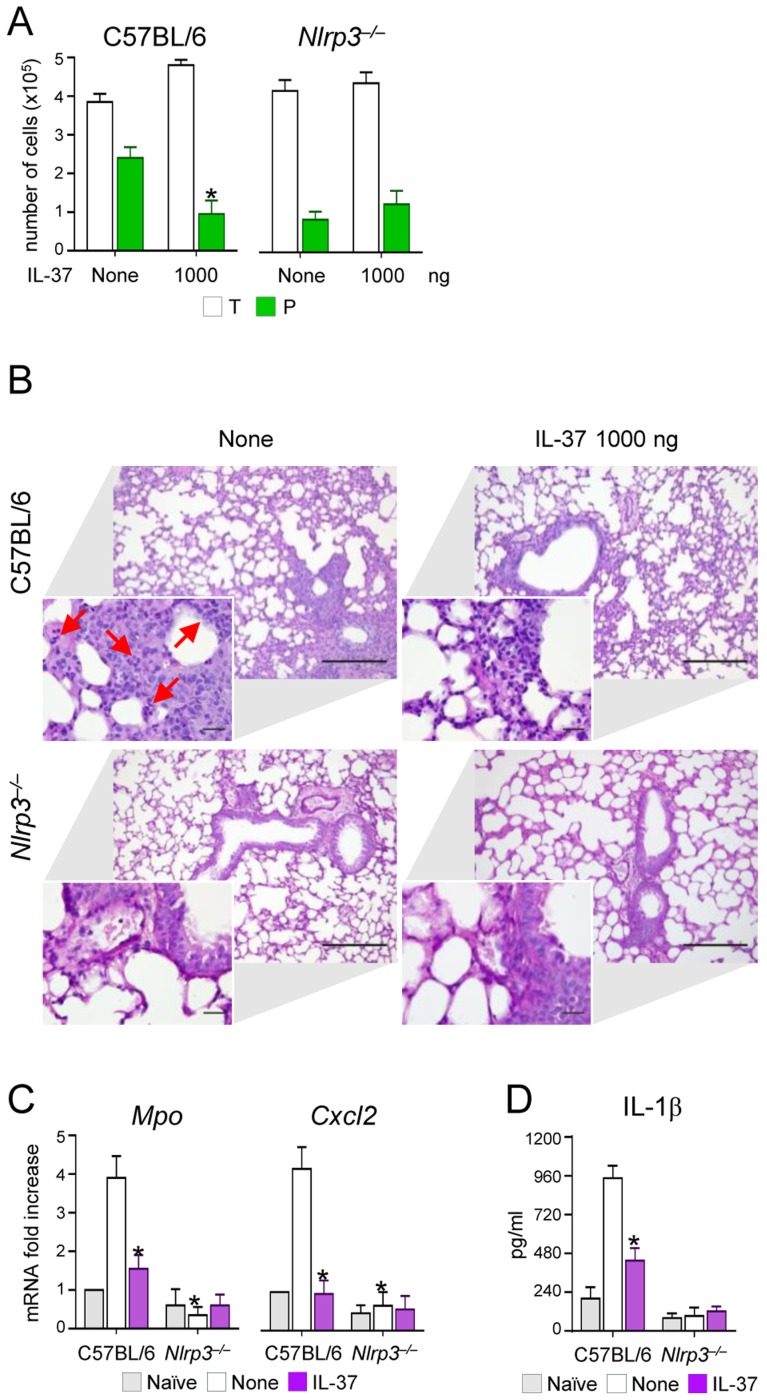
NLRP3-deficient mice exhibit reduced neutrophil recruitment and IL-1β production in pulmonary aspergillosis. C57BL/6 and *Nlrp3^−/−^* mice were infected intranasally with *A. fumigatus* and treated with 1000 ng/mouse recombinant IL-37 precursor administered intraperitoneally 1 hour before the infection. (**A**) BAL fluid morphometry [number of total (T) cells and polymorphonuclear neutrophils (P). Values represent the mean±SD of three mice per group and are representative of 3 independent experiments]. (**B**) Lung histology (periodic acid-Schiff staining) and cell recruitment (insets). Scale bars, 100 µm and 25 µm, respectively. Arrows indicate neutrophils. (**C**) *Mpo* and *Cxcl2* mRNA expression by RT-PCR on total lung cells. (**D**) IL-1β production (ELISA on lung homogenates). Assays were done 3 days after the infection. Data are representative (histology) or pooled from three experiments. *P<0.05, *Nlrp3^−/−^ vs* C57BL/6 mice and treated *vs* untreated (None) mice. Naïve, uninfected and untreated mice.

However, the induction of IL-10 did not apparently account for the anti-inflammatory activity of IL-37, as IL-37 still retained its effects in IL-10-deficient mice (Figure S2). This finding is in line with what described in experimental colitis in which an antibody to the IL-10 receptor did not affect the anti-inflammatory properties of transgenic mice expressing human IL-37 [13]. Thus, these data indicate that IL-37 may limit the recruitment of inflammatory neutrophils and damage in infected lungs by dampening NLRP3 inflammasome activation. To directly prove this, we assessed NLRP3-deficient mice for susceptibility to aspergillosis and the effects of IL-37 administration. The results showed that neutrophil recruitment in the BAL (Figure 3A) and lungs (Figure 3B), *Mpo* and *Cxcl2* expression (Figure 3C) and IL-1β production (Figure 3D) were lower in NLRP3-deficient than wild-type mice and were not modified by IL-37 treatment. These data suggest that one mechanism by which IL-37 exerts its anti-inflammatory effects in lung aspergillosis is by inhibition of NLRP3 inflammasome activity.

### IL-37 dampens inflammatory pathways in phagocytic cells

**Figure 4:**
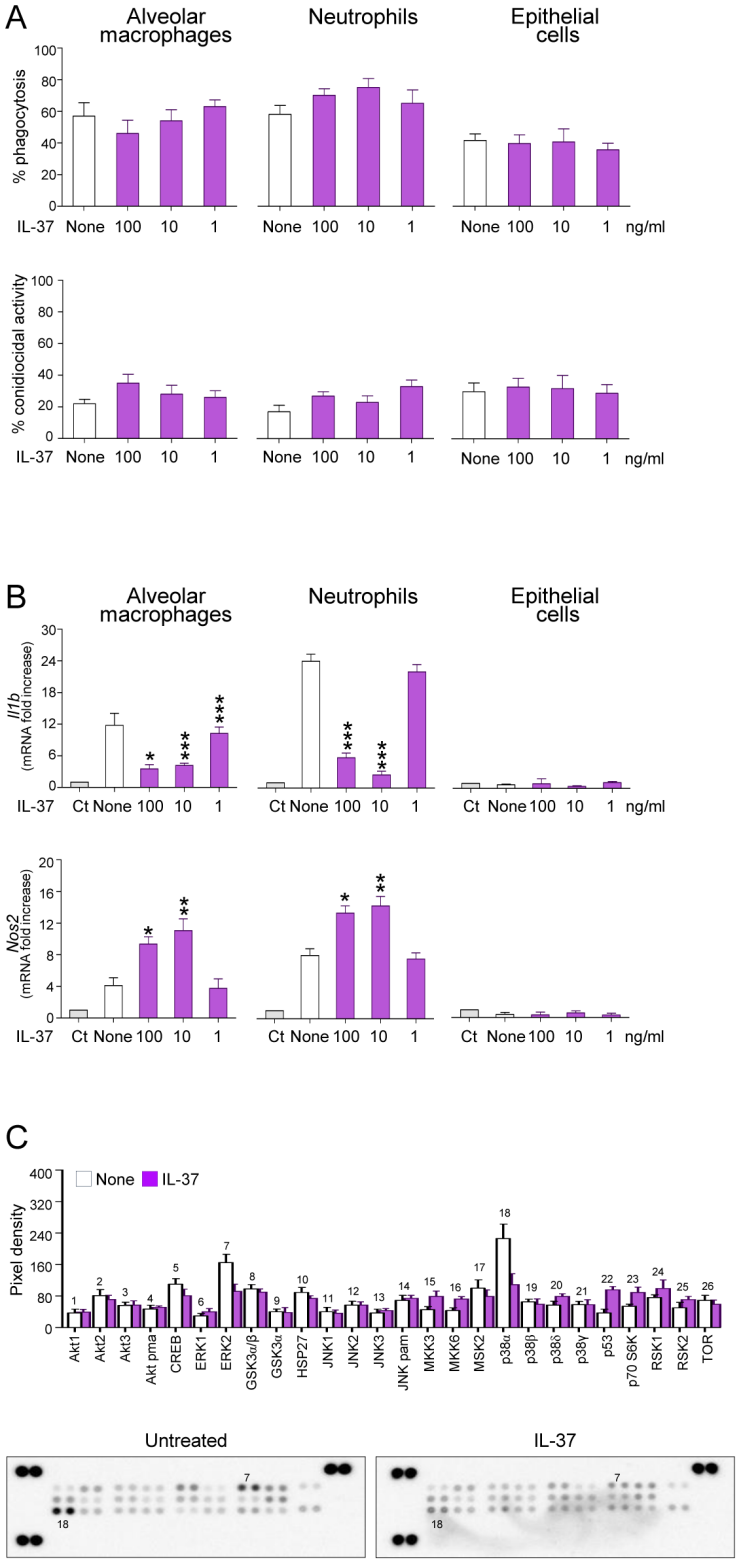
IL-37 dampens inflammasome activation in phagocytic cells. Alveolar macrophages and epithelial cells from naive mice and peripheral neutrophils were pre-exposed to recombinant IL-37 precursor for 8 hours before stimulation with live *Aspergillus* conidia for 2 hours. (**A**) Percent of phagocytosis and conidiocidal activity. (**B**) *Il1b* and *Nos2* mRNA expression by RT-PCR on total lung cells. Ct, control cells. None, *Aspergillus*-pulsed, untreated cells. (**C**) Activation of distinct intracellular kinases in RAW cells, using Proteome Profiler Array, pre-exposed to 100 ng/ml IL-37 for 8 hours before stimulation with live *Aspergillus* conidia for 30 min. Data are representative (Proteome Profiler Array) or pooled from two experiments. *P<0.05,**P<0.01, ***P<0.001, IL-37-stimulated *vs* unstimulated cells.

In order to identify which cell type is responsive to IL-37, we assessed the ability of IL-37 to affect the expression of *Il1b* in purified alveolar macrophages, lung epithelial cells and peripheral neutrophils from naïve mice in response to *Aspergillus* conidia. We did not observe that IL-37 decreased phagocytic capacity and fungicidal activity of phagocytes in vitro (Figure 4A), nevertheless recombinant IL-37 inhibited the expression of *Il1b* in response to conidia, in both macrophages and neutrophils (Figure 4B). Epithelial cells poorly responded to conidia stimulation, with and without IL-37 (Figure 4B). Of interest, IL-37 induced the expression of the inducible nitric oxide (*Nos2*), known to suppress inflammasome activation [27] and Th17 development [28]. IL-37 inhibits MAP kinase p38α in the human monocytic THP1 cell line [10]; therefore, we measured the phosphorylation of 19 kinases in the murine leukemic monocyte macrophage cell line RAW 264.7 pretreated with IL-37 and exposed to *Aspergillus* conidia, known to trigger the phosphorylation of p38 [29]. IL-37 greatly reduced p38α phosphorylation and, to a lesser extent, ERK1/2 phosphorylation in response to conidia (Figure 4C). These results indicate that IL-37 affects signal transduction pathways in response to conidia, likely impacting *Il1b* and *Nos2* gene expression.

### IL-37 fails to inhibit inflammasome activation in TIR-8/SIGIRR-deficient mice

**Figure 5:**
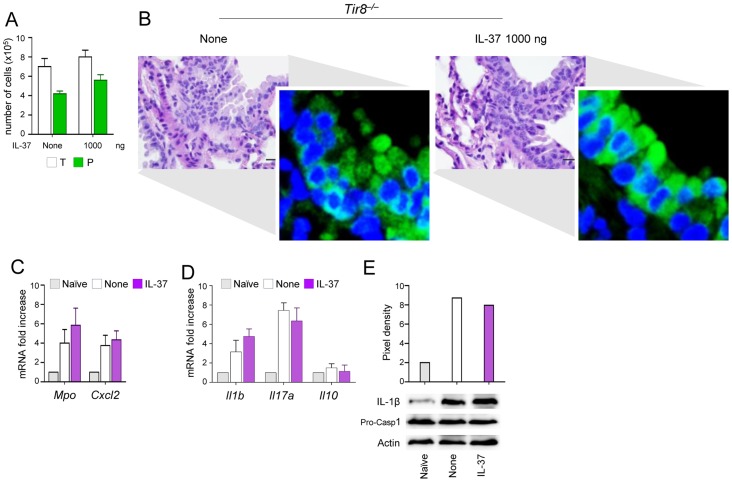
IL-37 fails to inhibit inflammasome activation in *Tir8^−/−^* mice. *Tir8^−/−^* mice were infected intranasally with *A. fumigatus* and treated intraperitoneally with recombinant IL-37 precursor, at the dose of 1000 ng/mouse, 1 hour before the infection. (**A**) BAL fluid morphometry [number of total (T) cells and polymorphonuclear neutrophils (P). Values represent the mean±SD of three mice per group and are representative of 3 independent experiments]. (**B**) Lung histology (PAS-stained sections). Scale bars, 25 µm. In the insets, NLRP3 expression by immunofluorescence staining of epithelial cells. Images were acquired using a fluorescence microscope with a 40× objective. Nuclei were counterstained with DAPI. (**C**) *Mpo* and *Cxcl2* mRNA expression and (**D**) cytokine gene expression (RT-PCR) on total lung cells. (**E**) Immunoblot with rabbit polyclonal IL-1β-specific antibody on whole lung lysates. Assays were done 3 days post-infection. Data are representative (histology) or pooled from two experiments. None, untreated mice. Naïve, uninfected and untreated mice.

We reported that TIR-8/SIGIRR is required for host resistance to fungal infections by reducing IL-1β–dependent activation of inflammatory Th17 responses [20]. In the present study, we assessed whether the inhibitory activity of recombinant IL-37 would require TIR-8/SIGIRR. To this purpose, we evaluated the impact of IL-37 on inflammasome activation and inflammation in *Tir8* ^−/−^ mice with aspergillosis. Consistent with previous findings [20], increased BAL neutrophilia (Figure 5A) and lung inflammation (Figure 5B) were observed in *Tir8* ^−/−^ mice along with an heightened expression of NLRP3 (Figure 5B inset). Consistently, *Mpo*, *CxCl2* (Figure 5C), *Il1b* and *Il17a* (Figure 5D) expression as well as the cleavage of the IL-1β precursor (Figure 5E) were all up-regulated in these mice. Treatment with 1000 ng/mouse of IL-37 one h before the infection neither limited inflammatory cell recruitment nor inhibited the heightened NALP3 expression activation in these mice (Figure 5B–E). IL-37 also failed to restore *Il10* expression in these mice (Figure 5D). These data support the concept that TIR-8/SIGIRR signaling is required for the anti-inflammatory effects of IL-37.

### IL-37 limits inflammation in fungal allergy and mice with cystic fibrosis

**Figure 6:**
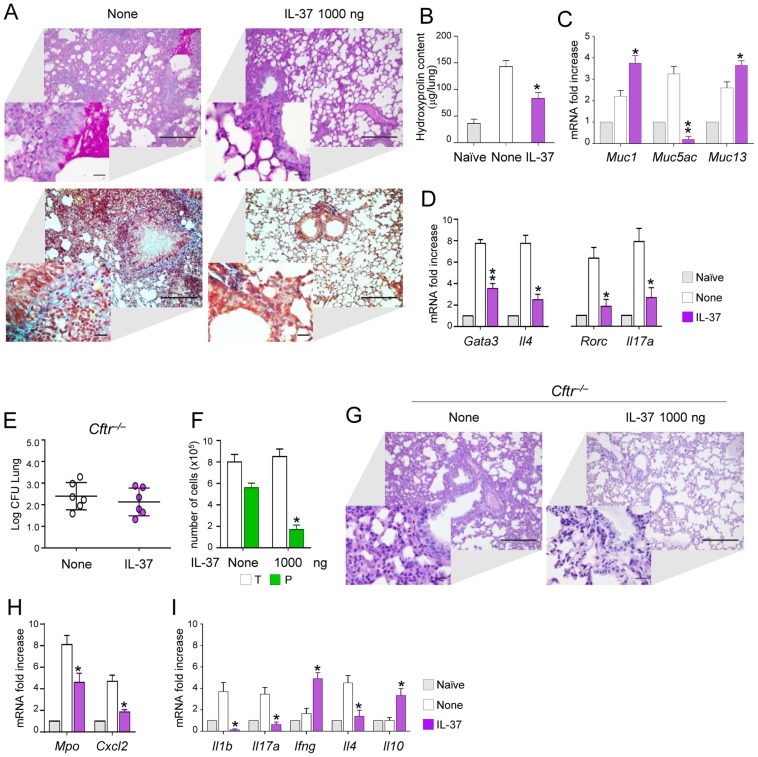
IL-37 restrains inflammation in fungal allergy and *Cftr^−/−^* mice. (**A**) Lung histology (PAS- and Masson's trichrome-stained sections, scale bars 100 and 25 (insets) µm); (**B**) hydroxyproline content (µg/lung); (**C**) expression of mucins (RT-PCR on total lung cells); (**D**) expression of cytokines and Th transcription factors in total lung cells from mice with ABPA and treated with IL-37. None, untreated mice. Naïve, uninfected and untreated mice. (**E**) Fungal growth (Log_10_ CFU, mean±SD) in the lungs of *Cftr^−/−^* mice infected intranasally with *A. fumigatus* and treated intraperitoneally with IL-37, at the dose of 1000 ng/mouse, 1 hour before the infection. (**F**) BAL fluid morphometry [number of total (T) cells and polymorphonuclear neutrophils (P) upon May Grunwald Giemsa staining]. (**G**) Lung histology (periodic acid-Schiff staining) and cell recruitment (insets). Scale bars, 100 µm and 25 µm in the insets; (**H**) *Mpo* and *Cxcl2* mRNA expression and (**I**) cytokine gene expression on total lung cells by RT-PCR, 3 days after the infection. Data are pooled from two experiments. *P<0.05, **P<0.01, treated *vs* untreated (None) mice. Naïve, uninfected and untreated mice.

To evaluate the potential for IL-37 to limit inflammation also in *Aspergillus* allergy, we resorted to a murine model of allergic bronchopulmonary aspergillosis (ABPA) in which both the Th2 and Th17 cell responses contribute to the inflammatory response [30]. Mice were sensitized to *Aspergillus* antigens and concomitantly treated with 1000 ng/mouse of IL-37. We found that IL-37 reduced mucin production (Figure 6A) and peribronchial fibrosis due to collagen deposition as shown by Masson's trichrome staining (Figure 6A) and hydroxyprolin content (Figure 6B). In addition, IL-37 markedly reduced the expression of the mucin Muc-5/5ac gene, a known marker of globlet cells metaplasia in murine airways [9]. In addition, IL-37 greatly decreased inflammatory cell recruitment and Th2/Th17cell activation (Figure 6D), a finding indicating that IL-37 has the potential to impact on the adaptive immune response. To evaluate the anti-allergic activity of IL-37 in a clinically relevant model, we resorted to *Cftr tm1Unc* (*Cftr^−/−^*) mice that are considered to mimic, to some extent, the airway inflammation and infection of human cystic fibrosis (CF) [31], [32]. We have already shown that *Cftr* ^−/−^ mice are highly susceptible to *Aspergillus* infection and allergy, due to an heightened inflammatory Th17/Th2 response [33]. *Cftr* ^−/−^ mice were pretreated with 1000 ng/mouse IL-37 one h before the infection and parameters of infection and inflammation were evaluated one day after the infection. We observed that IL-37 pretreatment, while not affecting the fungal burden (Figure 6E), decreased the numbers of neutrophils in BAL (Figure 6F) and lungs (Figure 6G) associated with lower expression of *Mpo* and *Cxcl2* (Figure 6H). IL-37 also decreased the expression *Il1b*, *Il17a*, *Il4* and increased that of *Il10* and *Ifng* (Figure 6I). These data point to IL-37 as a potent regulator of inflammation during respiratory fungal infection and allergy.

## Discussion

This study is the first to show the activation of the NLRP3 inflammasome in vivo during *A. fumigatus* infection and its inhibition by recombinant IL-37 precursor. Members of the inflammasome family are key players in host defense against C*andida albicans* [34]–[36] and control fungal opportunism and pathogenicity [37]. Activation of NLRP3 has also been shown in response to *A. fumigatus* in vitro [21], but a functional role in infection has not been demonstrated. Here, we observed that the activation of the NLRP3 inflammasome is associated with increased secretion of IL-1β and chemokines that mediate neutrophil recruitment into the lung. Although neutrophils serve potent antifungal effector function [38], in conditions of non-resolving inflammatory responses, neutrophils drive detrimental inflammation. Indeed, in such settings, mice deficient in IL-1R1 are protected [22], [38] and mice with hyper-functioning of IL-1β signaling have detrimental inflammatory responses [20]. Therefore, inflammasome activation and IL-1β secretion can drive pathological sequelae during *Aspergillus* infection. Although overexpression of IL-37 reduces IL-1beta secretion [10], the capacity of IL-37 to inhibit NLRP3 activation and IL-1β-mediated chemokine production described here impacts directly on inflammatory cell recruitment in the lung and on the Th balance. IL-37 decreased tissue damage during infection, a finding suggesting that regulation of inflammatory cell recruitment is essential to maintain normal tissue function. The ability to limit inflammatory cell recruitment was also observed in response to LPS, a finding consistent with the ability of IL-37 to reduce LPS-induced pro-inflammatory cytokine expression [10] and further pointing to a protective effect for IL-37 in respiratory infections.

The immunomodulatory action of IL-37 appears to occur at the level of myeloid cells, likely bone marrow-recruited macrophages and neutrophils, whose transcriptional program in response to conidia was indeed modified in the presence of recombinant IL-37. IL-37 increased *Nos2* gene expression, a finding that may suggest an impairment of the fungicidal activity of effector phagocytes by IL-37. However, we did not observe decreased phagocytic capacity or impaired fungicidal activity of phagocytes in vitro in the presence of IL-37. IL-37 increased the expression of β-defensins and cathelicidin, which may play a role in antifungal host defense [39], but the contribution of these peptides to the control of fungal growth by IL-37 is presently unknown.

Regardless of the specific downstream signaling pathways mediating the effects of IL-37, TIR8/SIGIRR is required. The Toll IL-1 Receptor (TIR) domain of SIGIRR has two mutations which likely act as decoys for MyD88 activation for IL-1 as well as TLR signaling. TIR-8 inhibits signaling receptor complexes of IL-1 family members associated with Th1 (IL-18), Th2 (IL-33) and Th17 (IL-1) and induces tolerogenic responses [40]. Thus, TIR-8/SIGIRR emerges as a non-redundant receptor for dampening inflammation and tissue damage in respiratory infections [20], [40]. In fungal infections, signaling through TIR-8/SIGIRR was required for the prevention of lethal inflammatory pathology associated with disregulated IL-1-dependent Th17 responses [20], [41].

Little is known about the interaction between TIR-8/SIGIRR and other members of the superfamily [42]. IL-37b may recruit TIR8/SIGIRR to activate the anti-inflammatory pathway [11]. Our study clearly shows that TIR-8/SIGIRR is involved in the anti-inflammatory activity of IL-37, but the molecular mechanisms of this interaction remains to be fully elucidated.

In the era in which damage control more than pathogen control has been suggested to provide new approaches for the treatment of infections and other diseases [43], our study seems to qualify IL-37 as an endogenous mediator of tissue tolerance during acute *Aspergillus* infection, likely including that occurring in the setting of neutrophil recovery. Moreover, the ability of IL-37 to also affect the development of adaptive immunity may suggest that recombinant IL-37 could be of benefit in conditions of non-resolving detrimental inflammation during acute *Aspergillus* infection but also fungal allergy.

## Materials and Methods

### Ethics statement

Murine experiments were performed according to the Italian Approved Animal Welfare Assurance A-3143-01 and Legislative decree protocol number 245/2011-B regarding the animal license obtained by the Italian Ministry of Health lasting for three years (2011–2014). Infections were performed under avertin anesthesia and all efforts were made to minimize suffering. The experimental protocol was designed in conformity with the recommendations of the European Economic Community (86/609/CEE) for the care and the use of laboratory animals, was in agreement with the Good Laboratory Practices and was approved by the animal care Committee of the University of Perugia (Perugia, Italy).

### Mice

Six to eight-week C57BL/6 (wild-type) female mice were purchased from Charles River (Calco, Italy). Genetically engineered homozygous *Cftr^−/−^* mice [44] were bred at the Cystic Fibrosis core animal facility at San Raffaele Hospital, Milan, Italy. *Tir8^−/−^* mice were raised on a 129/Sv and C57BL/6J mixed genetic background. *Il10^−/−^* mice were bred at the Animal Facility of the University of Perugia, Perugia, Italy. *Nlrp3^−/−^* mice were obtained from Francis Derouet, Université de Lausanne, Switzerland.

### Recombinant human IL-37

Full-length human IL-37 precursor with amino acids 1–218 (IL-37B isoform), was inserted in pCACTUS with a chicken beta actin promoter and N-terminal 6-histidines. After expression in *E. coli*, the recombinant molecule was purified on Talon followed by FPLC size exclusion. The peak isolated from the FPLC was applied to a C6 HPLC column and the IL-37 peak eluted in acetonitrile, isolated and lyophilized. The lyophilized IL-37 was reconstituted in PBS. On silver PAGE, recombinant IL-37 appears as a single band with a MW of 34 kDa.

### Fungal infection, allergy and treatment

Viable conidia from the *A. fumigatus* Af293 strain were obtained as described [30]. Mice were anesthetized in a plastic cage by inhalation of 3% isoflurane (Forane Abbot) in oxygen before intranasal instillation of 2×10^7^ resting conidia/20 µl saline. Recombinant human IL-37 was given intraperitoneally at different times (96, 48 and 1 hour) and at different concentrations (1000, 100, 10 and 1 ng/mouse) before the infection. Controls received the diluent alone. Mice were monitored for cell recruitment in the bronchoalveolar lavage fluid (BAL), histopathological analysis and chemokine or cytokine expression and production. For allergy, mice received an i.p. and s.c. injection of 100 µg of *A. fumigatus* culture filtrate extract (CCFA) dissolved in incomplete Freund's adjuvant (Sigma-Aldrich) followed by two consecutive intranasal injections (a week apart) of 20 µg CCFA. A week after the last intranasal challenge, mice received 10^7^ *Aspergillus* resting conidia and evaluated a week later [30]. IL-37 (1000 ng) was administered in concomitance with CCFA sensitization and *Aspergillus* inoculation. Ultrapure LPS from *Salmonella minnesota* Re 595 (Sigma-Aldrich) was given intranasally at the concentration of 10 µg/mouse. For histology, paraffin-embedded tissues were stained with Periodic acid-Schiff (PAS) and with Masson's trichrome staining to investigate the collagen deposition. Photographs were taken using a high- resolution Olympus DP71 microscope.

### Collection of BAL

Lungs were filled thoroughly with 1.0 ml aliquots of pyrogen-free saline through a 22-gauge bead-tipped feeding needle introduced into the trachea. BAL fluid was collected in a plastic tube on ice and centrifuged at 400× g at 4°C for 5 min. For differential BAL fluid cell counts, cytospin preparations were stained with May-Grünwald Giemsa reagents (Sigma-Aldrich). At least 10 fields (200 cells/field) were counted, and the percent of polymorphonuclear (PMN) and mononuclear (MNC) cells was calculated [45]. Photographs were made using a high-resolution Olympus DP71 microscope.

### Terminal deoxynucleotidyl transferase-mediated deoxyuridine triphosphate nick-end labeling (TUNEL) of lung sections

The lungs were fixed in 4% buffered paraformaldehyde, pH 7.3, for 36 h and embedded in paraffin. Sections were de-paraffinized, re-hydrated and treated with 0.1 M citrate buffer, pH 6.0, for 20 min in a water bath, washed and blocked in 0.1 M Tris/HCl buffer, pH 7.5, supplemented with 3% bovine serum albumin and 20% FCS. The slides were then incubated with fluorescein-coupled dUTP and TUNEL enzyme (Roche Diagnostics) in the presence of terminal deoxynucleotidyl transferase. The samples were then washed with PBS, incubated for 10 min at 70°C to remove unspecific binding. The sections were mounted and analyzed by fluorescent microscopy using a 40× objective.

### Immunofluorescence

The lung was removed and fixed in 10% phosphate-buffered formalin, embedded in paraffin and sectioned at 5 µm. Sections were then rehydrated and after antigen retrieval in citrate buffer (10 mM, pH 6), sections were blocked with 5% BSA in PBS and stained with goat-anti-CIAS1/Nlrp3 antibody overnight at 4°C followed by donkey anti-goat IgG H&L (DyLight 488) secondary antibody (both from Abcam). Images were acquired using a fluorescence microscope (BX51 Olympus) with a 20× objective and the analySIS image processing software (Olympus). 4′-6-Diamino-2-phenylindole (DAPI, Molecular Probes, Invitrogen) was used to counterstain tissues and to detect nuclei.

### Cell preparation, phagocytosis, conidiocidal activity and culture

Alveolar macrophages were isolated from total lung cells after 2-hour plastic adherence at 37°C. Murine CD11b^+^ Gr-1^+^ neutrophils were positively selected with magnetic beads (Miltenyi Biotech) [46] from the peritoneal cavity of uninfected wild-type mice 8 h after the intraperitoneal injection of 1 ml endotoxin-free 10% thioglycollate solution. Endotoxin was depleted from all solutions with Detoxi-gel (Pierce). On fluorescence-activated cell sorting (FACS) analysis, Gr-1^+^ neutrophils were >98% pure and stained positive for the CD11b myeloid marker. Lung epithelial cells were isolated as described [39]. Cells were pre-exposed to IL-37 for 8 hours before stimulation with live *Aspergillus* conidia for 2 hours for the assessment of phagocytosis, conidiocidal activity and cytokine gene expression.

### Whole proteomic RAW analysis

RAW 264.7 cells (ATCC) were exposed to 100 ng/ml IL-37 precursor for 8 hours before stimulation with live *Aspergillus* conidia at a cell/fungi ratio of 1∶1, for 30 minutes. The relative phosphorylation of 26 Phospho-Mitogen-activated Protein Kinase (MAPK) was performed using the Proteome ProfilerArray (R&D Systems). Kinases were captured by 26 different antibodies spotted in duplicate on a nitrocellulose membrane. Levels of phosphorylated protein were then assessed using phospho-specific antibodies and chemiluminescent detection.

### Western blot analysis

An equal amount of whole lung tissue were lysed in 2× Laemli buffer and separated in 14% Tris/glicine SDS gel, transferred to a nitrocellulose membrane, probed with rabbit anti-mouse IL-1β (Biolegend) or rabbit anti-caspase-1-p10 (Santa Cruz). Goat anti-rabbit IgG-HRP (Sigma-Aldrich) was used as secondary antibodies. Normalization was performed on rabbit anti-actin antibody (Santa Cruz) and quantification was obtained by densitometric image analysis using Image Lab 3.1.1 software (Bio-Rad) as previously described [47].

### Flow cytometry analysis

All staining reactions were performed at 4°C on cells first exposed to Fc receptor mAb (2.4G2) in order to reduce nonspecific binding. Anti CD11b (M1/70) and anti-CD11c (N418) were purchased from BD Biosciences-Pharmingen. Cells were analyzed with a BD LSRFortessa flow cytometer (BD) equipped with BD FACSDiva 7.0 software.

### ELISA and real-time PCR

The levels of cytokines in lung homogenates were determined by mouse ELISAs (R&D Systems). The detection limits of the ELISAs were less than 3 pg/ml for IL-10 and 5 pg/ml for IL-1β, respectively. Real-time RT-PCR was performed using the Stratagene Mx3000P QPCR System and SYBR Green chemistry (Stratagene). Cells were lysed and total RNA was reverse transcribed with cDNA Synthesis Kit (BioRad), according to the manufacturer's instructions. The PCR primers were as listed in Table 1. Amplification efficiencies were validated and normalized against GAPDH. The thermal profile for SYBR Green real-time PCR was at 95°C for 3 min, followed by 40 cycles of denaturation for 30 s at 95°C and an annealing/extension step of 30 sec at 60°C. Each data point was examined for integrity by analysis of the amplification plot. The mRNA-normalized data were expressed as relative gene mRNA in treated compared to untreated experimental groups or cells.

**Table 1:** Real-time murine PCR primers used in this study.

Gene name	Primer sequence
*Mpo*	Forward, 5′-TTACACCCCAGGCATAAAAA-3′
	Reverse, 5′-TTCCATACAGCTCAGCACAA-3′
*Cxcl1*	Forward, 5′-CCGCTCGCTTCTCTGTGC-3′
	Reverse, 5′-CTCTGGATGTTCTTGAGGTGAATC-3′
*Cxcl2*	Forward, 5′-CCAACCACCAGGCTACAG-3′
	Reverse, 5′-CTTCAGGGTCAAGGCAAAC-3′
*Gata3*	Forward, 5′-TCTGGAGGAGGAACGCTAATG-3′
	Reverse, 5′-GGCTGGAGTGGCTGAAGG-3′
*Rorc*	Forward, 5′-ACAACAGCAGCAAGTGATGG-3′
	Reverse, 5′-CCTGGATTTATCCCTGCTGA-3′
*Tnfa*	Forward, 5′-CGAGTGACAAGCCTGTAGCC-3′
	Reverse, 5′-AAGAGAACCTGGGAGTAGACAAG-3′
*IL6*	Forward, 5′-CCGGAGAGGAGACTTCACAG-3′
	Reverse, 5′-TCCACGATTTCCCAGAGAAC-3′
*Ifng*	Forward, 5′-ACTGGCAAAAGGATGGTGAC-3′
	Reverse, 5′-TGAGCTCATTGAATGCTTGG-3′
*Il17a*	Forward, 5′-GACTACCTCAACCGTTCCAC-3′
	Reverse, 5′-CCTCCGCATTGACACAGC-3′
*Il4*	Forward, 5′-CGGCATTTTGAACGAGGTCACAGG-3′
	Reverse, 5′-AGCACCTTGGAAGCCCTACAGACG-3′
*Il10*	Forward, 5′-CCCTTTGCTATGGTGTCCTT-3′
	Reverse, 5′-TGGTTTCTCTTCCCAAGACC-3′
*Nalp3*	Forward, 5′-ATGCTGCTTCGACATCTCCT-3′
	Reverse, 5′-GTTTCTGGAGGTTGCAGAGC-3′
*Il1b*	Forward, 5′-TGACGGACCCCAAAAGATGAAGG-3′
	Reverse, 5′-CCACGGGAAAGACACAGGTAGC-3′
*Il1a*	Forward, 5′-CTGCAGTCCATAACCCAT-3′
	Reverse, 5′-TGACAAACTTCTGCCTGACG-3′
*Il1ra*	Forward, 5′-TTGTGCCAAGTCTGGAGATG-3′
	Reverse, 5′-CAGCTGACTCAAAGCTGGTG-3′
*p47*	Forward, 5′- TAGAGACTCCTCCCATGCCT -3′
	Reverse, 5′- CACTGCCTCCTCTCATGCTA -3′
*p67*	Forward, 5′- CTATCTGGGCAAGGCTACGGTT -3′
	Reverse, 5′- CACAAAGCCAAACAATACGCG -3′
*gp91*	Forward, 5′-AAAGGAGTGCCCAGTACCAAAGT-3′
	Reverse, 5′-TACAGGAACATGGGACCCACTAT-3′
*Nos2*	Forward, 5′-GGACACTGCCGCCAACATCTAC-3′
	Reverse, 5′-CACCCAAAGTGCTTCAGTCA-3′
*Muc1*	Forward, 5′-TGAGCCAGGACTTCTGGTAG-3′
	Reverse, 5′-CCTTCTGAGAGCCACCACTA-3′
*Muc5ac*	Forward, 5′-CTGGACCTGGAGGTTGTATG-3′
	Reverse, 5′-CAGTAGTGAGGGTTGGATGG-3′
*Muc13*	Forward, 5′-ACATGGTGAAGGGTCAAGAA-3′
	Reverse, 5′-AGATGAACTACCCACGGTCA-3′

### Hydroxyproline assay

The total collagen content of the lung tissue was measured spectrophotometrically by absorbance at 560 nm to quantify the lung hydroxyproline content 7 days after infection. Briefly, the minced lung lobes were homogenized in dH_2_O, using 100 µl H_2_O for every 10 mg of tissue. To a 100 µl of sample homogenates, add 100 µl concentrated 12 N HCl in a pressure-tight, teflon capped vial and hydrolyze at 120°C for 3 hours. After reaction with Chloramine T reagent (incubate at room temperature for 5 min) and DMAB reagent (incubate for 90 min at 60°C), the absorbance was measured at 560 nm. The results were expressed as µg hydroxyproline per mg of wet lung weight using a standard curve (0,1 mg/ml) (BioVision).

### Statistical analysis

Data are expressed as mean ± SD. Horizontal bars indicate the means. For multiple comparisons, p values were calculated by a one-way ANOVA (Bonferroni's post hoc test). For single comparison, p values were calculated by a twotailed Student's t test. The data reported are either from one representative experiment (histology, TUNEL and western blotting) or pooled otherwise. The in vivo groups consisted of 4–6 mice/group. Data were analyzed by GraphPad Prism 4.03 program (GraphPad Software).

## Supporting Information

Figure S1 **IL-37 reduces inflammation when administered after the infection.** C57BL/6 mice were infected intranasally with *A. fumigatus* and treated with 1000 ng/mouse IL-37 administered intraperitoneally for 3 consecutive days starting the day of the infection. Mice were assessed for: (**A**) number of total (T) cells and polymorphonuclear neutrophils (P) in the BAL. Values represent the mean±SD of three mice per group and are representative of 2 independent experiments; (**B**) lung histology (periodic acid-Schiff staining) and cell recruitment (insets). Scale bars, 100 µm and 25 µm in the insets; (**C**) myeloperoxidase (*Mpo*) and *Cxcl2* mRNA expression by RT-PCR on total lung cells. Assays were done a day after the last treatment. *P<0.05,**P<0.01, treated *vs* untreated (None) mice. Naïve, uninfected and untreated mice.
(TIF)

Figure S2 **The anti-inflammatory activity of IL-37 in not dependent on IL-10.** *Il10^−/−^* mice were infected intranasally with *A. fumigatus* and treated with 1000 ng/mouse IL-37 administered intraperitoneally 1 h before the infection. Mice were assessed for: (**A**) number of total (T) cells and polymorphonuclear neutrophils (P) in the BAL. Values represent the mean±SD of three mice per group and are representative of 3 independent experiments; (**B**) lung histology (periodic acid-Schiff staining) and cell recruitment (insets). Scale bars, 100 µm and 25 µm in the insets. Assays were done a day after the infection. *P<0.05, treated *vs* untreated (None) mice. Naïve, uninfected and untreated mice.
(TIF)
