# Supplementary figures and images for: IL-37 Inhibits Inflammasome Activation and Disease Severity in Murine Aspergillosis
Source: PLoS Pathog. 2014 Nov 6;10(11):e1004462. doi: 10.1371/journal.ppat.1004462 (PMC4223056; doi:10.1371/journal.ppat.1004462)

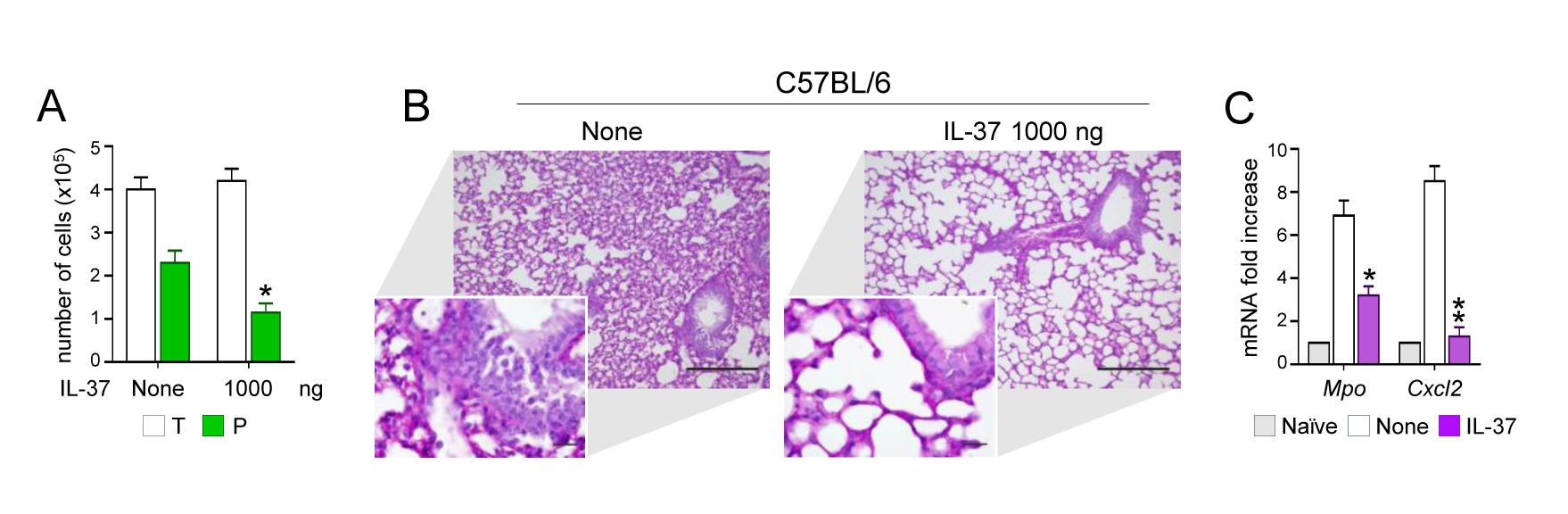

Supplement: Figure S1 — IL-37 reduces inflammation when administered after the infection. C57BL/6 mice were infected intranasally with A. fumigatus and treated with 1000 ng/mouse IL-37 administered intraperitoneally for 3 consecutive days starting the day of the infection. Mice were assessed for: (A) number of total (T) cells and polymorphonuclear neutrophils (P) in the BAL. Values represent the mean±SD of three mice per group and are representative of 2 independent experiments; (B) lung histology (periodic acid-Schiff staining) and cell recruitment (insets). Scale bars, 100 µm and 25 µm in the insets; (C) myeloperoxidase (Mpo) and Cxcl2 mRNA expression by RT-PCR on total lung cells. Assays were done a day after the last treatment. *P<0.05,**P<0.01, treated vs untreated (None) mice. Naïve, uninfected and untreated mice. (TIF) [file ppat.1004462.s001.tif]

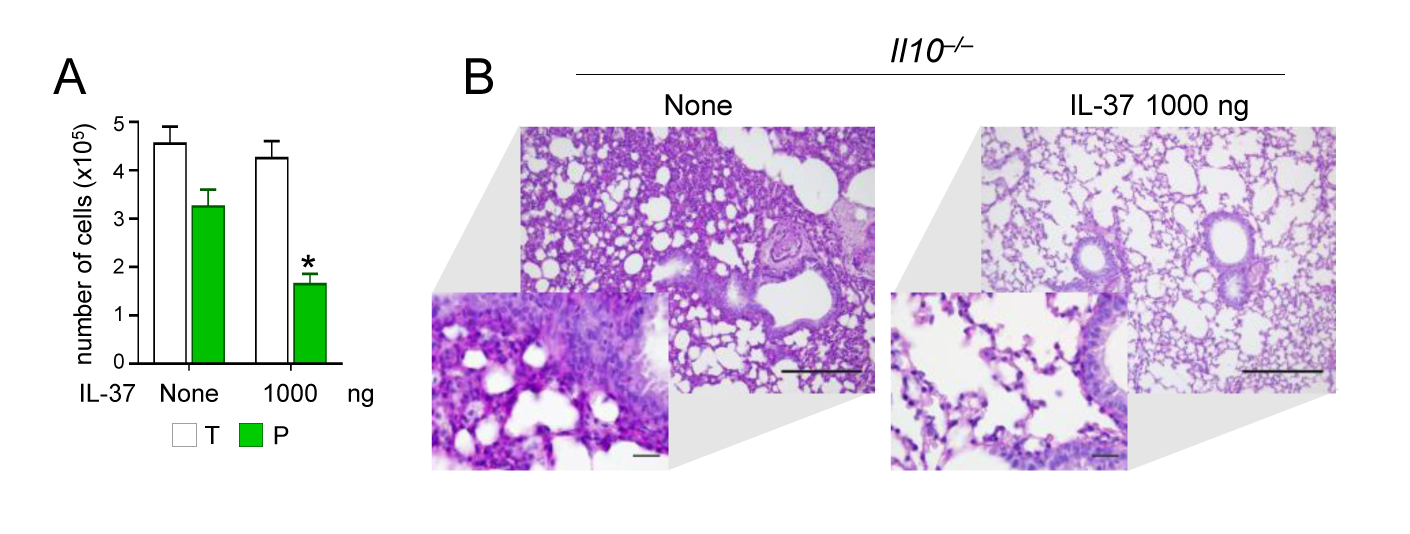

Supplement: Figure S2 — The anti-inflammatory activity of IL-37 in not dependent on IL-10. Il10−/− mice were infected intranasally with A. fumigatus and treated with 1000 ng/mouse IL-37 administered intraperitoneally 1 h before the infection. Mice were assessed for: (A) number of total (T) cells and polymorphonuclear neutrophils (P) in the BAL. Values represent the mean±SD of three mice per group and are representative of 3 independent experiments; (B) lung histology (periodic acid-Schiff staining) and cell recruitment (insets). Scale bars, 100 µm and 25 µm in the insets. Assays were done a day after the infection. *P<0.05, treated vs untreated (None) mice. Naïve, uninfected and untreated mice. (TIF) [file ppat.1004462.s002.tif]
